# Average Gait Differential Image Based Human Recognition

**DOI:** 10.1155/2014/262398

**Published:** 2014-05-06

**Authors:** Jinyan Chen, Jiansheng Liu

**Affiliations:** ^1^School of Computer Software, Tianjin University, Tianjin 300072, China; ^2^College of Science, Jiangxi University of Science and Technology, Ganzhou 330200, China

## Abstract

The difference between adjacent frames of human walking contains useful information for human gait identification. Based on the previous idea a silhouettes difference based human gait recognition method named as average gait differential image (AGDI) is proposed in this paper. The AGDI is generated by the accumulation of the silhouettes difference between adjacent frames. The advantage of this method lies in that as a feature image it can preserve both the kinetic and static information of walking. Comparing to gait energy image (GEI), AGDI is more fit to representation the variation of silhouettes during walking. Two-dimensional principal component analysis (2DPCA) is used to extract features from the AGDI. Experiments on CASIA dataset show that AGDI has better identification and verification performance than GEI. Comparing to PCA, 2DPCA is a more efficient and less memory storage consumption feature extraction method in gait based recognition.

## 1. Introduction

With the development of information and Internet technology, it is very necessary to authenticate and authorize human securely. The rapid growth of e-commerce also needs a reliable identification method to ensure safety transaction. As a promising authentication method, biometrics is attracting more and more attention. Biometrics overcomes the inherent flaws and limitations of conventional identification technology and brings a highly secure identification and authentication method. Traditional biometrical resources include fingerprint, face, and iris, which have been widely used for authentication. However, these biometrical features have the following disadvantage. (1) These features cannot be taken in a relative long distance. (2) User's cooperation is required to get good results. As a new biometrics method, gait based human identification overcomes the above limitation and is attracting more and more researchers.

Human gait is the manner of one walking, which was firstly studied in medical field. Doctors analyzed the human gait to find out whether patients had health problems [1, 2]. Later researchers found that just like fingerprint and iris, almost everyone has his distinctive walking style [3, 4]. So it was believed that gait could also be used as a biological feature to recognize the person. Although suffering from clothing, shoes, view angel, or environmental context, human gait is still a promising identification method.

Human walking can be considered as an images sequence; however, most of the current model-free gait based identification methods extract features from image sequence without considering its contained spatiotemporal information. The method proposed in this paper focuses on the difference among the images sequence while constructing the feature image. The procedure can be described as follows. The silhouettes were normalized to the same height and aligned by the centroid. Then the difference between two adjacent silhouettes was accumulated to get the average gait differential image (AGDI) which is used as the feature image of one walking. Two-dimensional principal component analysis is used to extract feature from AGDI.

## 2. Related Work and Our Contribution

Usually recognition based on human gait includes several different approaches like walking, running, and jumping. In this paper we would like to restrict the recognition to walking. Currently human gait recognition can be divided into two categories: model-based methods and motion-based ones.

Model-based approaches aim to describe human movement using a mathematical model. Cunado et al. [5] used Hough transform to extract the positions of arms, legs, and torso and then use articulated pendulum to match those moving body parts. Yoo et al. [6] divided the body into head, neck, waist, leg, and arm by image segmentation and then got the moving curves of these body parts, respectively. Lee and Grimson [7] used 7 ellipses to model the human body and applied the ellipses' movement features to identify human. Yam et al. [8, 9] used dynamically coupled oscillator to describe and analyze the walking and running style of a person. Tafazzoli and Safabakhsh [10] constructed movements model based on anatomical proportions; then, Fourier transform was used to analyze human walking.

Model-free methods focused on the statistics information derived from the human gait. Cheng et al. [11] took HMM and manifold to analyze the relationship between the human and their gait images. Chen et al. [12] used parallel HMM to describe the features of human gait. Kale et al. [13] used “frieze” patterns to get features from image sequence and use them to identify a human. Murase and Sakai [14] speeded up the comparison of human gait by parametric eigenspace representation. Little and Boyd [15] derived scale-independent scalar features from optical flow information of walking figures to recognize individuals. Wang et al. [16] extracted feature by unwrapping the outer contour of silhouette and use PCA to reduce the dimension of the feature. Lee et al. [17] adopted product of Fourier coefficients as a distance measure between contours to recognize gait. Hu [18] combined the enhanced Gabor (EG) representation of the gait energy image and the regularized local tensor discriminate analysis (RLTDA) method in human identification. Hong et al. [19] proposed probabilistic framework to identify a human. Wang et al. [20] proposed spatiotemporal information analysis to get the features of human walking. Collins et al. [21] extract key frames from the image sequence and compare the key frames similarity by normalized correlation. Sarkar et al. [22] estimated the similarity between the gallery image sequence and the probe image sequence by directly computing the correlation between the frame pairs. Chen [23] proposes image correlation based human identification method.

Our method is similar to gait energy image (GEI) proposed by Yu et al. [24], Han and Bhanu [25], and frame difference energy image (FDEI) proposed by Chen et al. [26]. The major difference lies in the approach to generate the feature image. GEI is obtained by directly adding up every normalized silhouette. FDEI is obtained by taking the difference from every adjacent two frames and then combined with the “denoised” GEI. In this paper the difference between every two frames will be accumulated to generate average gait differential image. We also enhanced the feature extraction method by using 2DPCA, which has been used in the application of face recognition [27].

In comparison with the works of state of the art, the contributions of this paper are as follows.

*Gait Representation Method.* We propose a new gait feature representation which is called average gait differential image. Comparing to GEI, our method has the advantage of better performance. 

*Feature Extraction Method.* Two-dimensional principal component analysis (2DPCA) is used to extract features from AGDI, which can be more efficient and save more storage comparing to the widely used one-dimensional principal component analysis (PCA).

## 3. Average Gait Differential Image (AGDI) Representation

### 3.1. The Construction of AGDI

The construction of average differential image can be expressed in the following steps.

*Silhouette Segmentation.* Gauss model is used to get the background model from the original images sequence. To eliminate the effect of noise, every image is blurred by Gauss filter. The method proposed by Wang et al. [16] is used to extract walking object from the original images. 

*Normalization.* To exclude the distance effect, every silhouette is normalized to the same height using bicubic interpolation. 

**Figure 1:**
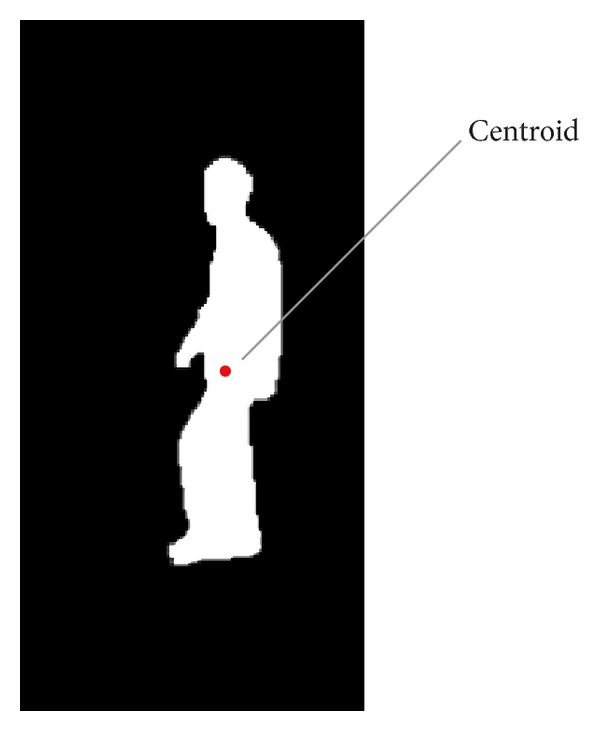
The centroid of a silhouette.

*Alignment and Subtraction.* We define the centroid (*x*_*c*_, *y*_*c*_) of a silhouette as follows:

(1) xc=1n∑i=1nxi,yc=1n∑i=1nyi,

where *n* is the number of pixels in the silhouette.  Figure 1 shows the centroid of a silhouette.

Suppose that *I*_*j*_ and *I*_*j*+1_ are two adjacent images aligned by the centroid; the gait differential image *D*_*j*_ can then be defined as follows:

(2) $$\begin{array}{c} D_{j}\left(x,y\right)=\{\begin{array}{cc} 0\;\;\text{if}\;\;I_{j}\left(x,y\right)=I_{j+1}\left(x,y\right) & \\ 1\;\;\text{if}\;\;I_{j}\left(x,y\right)\neq I_{j+1}\left(x,y\right), & \end{array} \end{array}$$

where *j* is the frame number in the image sequence and *x* and *y* are values in the 2D image coordinate.

**Figure 2:**
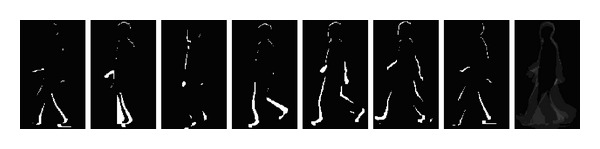
Differential images and average gait differential image.

*Get the Average Gait Differential Image.* By overlapping all the differential images of one human gait cycle, we can get the following average gait differential image:

(3) $$\begin{array}{c} G\left(x,y\right)=\frac{1}{N-1}\overset{N-1}{\underset{j=1}{\sum }}D_{j}\left(x,y\right), \end{array}$$

where *N* is the number of frames in the complete gait cycle(s) of a silhouette sequence.  Figure 2 shows the differential images in a gait cycle and the average gait differential image, respectively.

### 3.2. Feature Extraction

Although intheprevious section we have compressed the human gait features into one image, the dimensionality of the average gait differential image is still very large. The most commonly used dimensional reduction method is principal component analysis (PCA). In traditional PCA method, every two-dimensional image must be transformed into one-dimensional vector, leading to a covariance matrix with large size. This large matrix will use massive memory storage and is difficult to be evaluated accurately.

To reduce memory storage and speed up the calculation, this paper adopts the two-dimensional principal component analysis (2DPCA) to reduce the dimensionality, which was first proposed by Yang et al. [27] in the recombination of human face. Our final target is to project average gait differential image *G*, a *m* × *n* random matrix, onto a *m*-dimension projected vector *Y* which is called the projected feature vector of image *G* by the following linear transformation [27]:

(4) $$\begin{array}{c} Y=GW, \end{array}$$

where *W* denotes a *n*-dimensional unitary column vector. To preserve the features of *G*, *W* should make *Y* have the maximum scatter. We define *S*_*y*_ as the scatter of *Y* [27] as follows:

(5) Sy=E(Y−E(Y))(Y−E(Y))T=E(GW−E(GW))(GW−E(GW))T=E(G−E(G))WWT(G−E(G))T.

The trace of *S*_*y*_ can be expressed as

(6) $$\begin{array}{c} \mathrm{tr}\left(S_{y}\right)=W^{T}\left(E{\left(G-E\left(G\right)\right)}^{T}\left(G-E\left(G\right)\right)\right)W. \end{array}$$

Here, we can define the image covariance matrix as

(7) $$\begin{array}{c} C_{t}=E{\left(G-E\left(G\right)\right)}^{T}\left(G-E\left(G\right)\right). \end{array}$$

In this paper, average gait differential image for each individual (1,2,…, *M*) is expressed as *G*_1_, *G*_2_ ⋯ *G*_*M*_, and then *C*_*t*_ can be calculated by

(8) $$\begin{array}{c} C_{t}=\overset{M}{\underset{i=1}{\sum }}{\left(G_{i}-\overset{-}{G}\right)}^{T}\left(G_{i}-\overset{-}{G}\right). \end{array}$$

Our target is to find a series of *W*_opt_ in formula (6) to make tr (*S*_*y*_) have the maximum value. According to [13], the optimal projection axis *W*_opt_ is the unitary orthogonal eigenvector of *C*_*t*_ corresponding to the largest eigenvalue. We define the first *d* unitary orthogonal eigenvector as *W*_1_, *W*_2_,…, *W*_*d*_; that is,

(9) {W1,…Wd}=arg max⁡(WTCtW)WiWj=0, i≠j, i,j=1,…d,WiWj=1, i=j, j=1,…d.

The first d optimal projection vectors, *W*_1_,…, *W*_*d*_, are used to extract features from the average different images. That is to say, given an average gait differential image *X*, let

(10) $$\begin{array}{c} Y_{k}=GW_{k},\;k=1,2\ldots d. \end{array}$$

Then we get a series of projected feature vectors, *Y*_1_ ⋯ *Y*_*d*_, which are different from those scalar counterparts obtained from PCA. By using 2DPCA, the original *m* × *n* image is projected to a *m* × *d* (*d* ≤ *n*) feature matrix *Y* as

(11) $$\begin{array}{c} Y=\left[\begin{array}{c} Y_{1}\\ \vdots \\ Y_{d} \end{array}\right]. \end{array}$$

The distance between two feature matrixes is defined as

(12) $$\begin{array}{c} d\left(Y\left(i\right),Y\left(j\right)\right)=\overset{d}{\underset{k=1}{\sum }}||Y_{k}^{\left(i\right)}-Y_{k}^{\left(j\right)}||, \end{array}$$

where ||*Y*_*k*_^(*i*)^ − *Y*_*k*_^(*j*)^|| means the Euclidean distance between two vectors.

### 3.3. Identification and Verification

Following the pattern proposed by Sarkar et al. [22], we evaluate performance for both identification and verification scenarios.

In the scenario of identification, every images sequence in the gallery (training set) is transformed to a *m* × *d* feature matrix *Y*(*i*) by the method described in Section 3.2. Given a probe silhouette sequence, its transformed feature matrix is defined as *P*. This probe *P* is assigned to person *k* by using the nearest neighbor method:

(13) $$\begin{array}{c} d\left(Y\left(k\right),P\right)=\underset{i}{\min}\;d\left(Y\left(i\right)-P\right). \end{array}$$

In the scenario of verification, the similarity between two feature matrixes is defined as the negative of distance; that is,

(14) $$\begin{array}{c} \text{Sim}\left(Y\left(i\right),Y\left(j\right)\right)=-\overset{d}{\underset{k=1}{\sum }}||Y_{k}^{\left(i\right)}-Y_{k}^{\left(j\right)}||. \end{array}$$

In this paper the similarity between a probe, *P*_*j*_, and *Y*(*i*) in the gallery is defined as *z*-normed similarity [28]:

(15) $$\begin{array}{c} \text{Sim}\left(P_{j},Y\left(i\right)\right)=\frac{\text{Sim}\left(P_{j},Y\left(i\right)\right)-\text{Mea}{\text{n}}_{i}\text{Sim}\left(P_{j},Y\left(i\right)\right)}{\text{s}.\text{d}._{i}\text{Sim}\left(P_{j},Y\left(i\right)\right)}, \end{array}$$

where s.d. is standard deviation.

FAR (false acceptance rate), FRR (false rejection rate), and EER (equal error rate) are used to evaluate the performance of verification [22].

### 3.4. DPCA-Based Average Gait Differential Image Reconstruction

In PCA the principal components and eigenvectors can be combined to reconstruct the original matrix. Similarly, 2DPCA can also be used to reconstruct an average gait differential silhouette.

Suppose that the eigenvectors corresponding to the largest *d* eigenvectors of *C*_*t*_ are *W*_1_,…, *W*_*d*_; that is,

(16) $$\begin{array}{c} Y_{k}=GW_{k},\;k=1,\ldots ,d,\\ \left[Y_{1}\cdots Y_{d}\right]=X\left[W_{1}\cdots W_{d}\right]. \end{array}$$

According to formula (9), *W*_1_ ⋯ *W*_*d*_ are normal orthogonal vectors so the new reconstructed image $\tilde{G}$ can be expressed as

(17) G~=G[W1⋯Wd][W1⋯Wd]T=[Y1⋯Yd][W1⋯Wd]T=∑k=1dYkWkT.

## 4. Experiments and Analysis

### 4.1. Data and Parameters

CASIA gait database (Dataset B) [29], one of the largest gait databases in gait-research field, is used in the following experiment. Dataset B consists of 124 subjects (93 males and 31 females) captured from 11 view angles (ranging from 0 to 180° degree with view angle interval of 18). For every person there are six normal walking sequences (named normal-01⋯normal-06) conducted from every view angle. Every walking sequence contains 3–8 gait cycles (about 40–100 frames). The video frame size is 320 × 240 pixels, and the frame rate is 25 fps. We use all the 124 objects in Dataset B to carry out our experiments.

In all the following experiments, 2DPCA method was used to get features from the images and 20 eigenvectors corresponding to the first 20 eigenvalues are used to produce features (*d* = 20). The size of original image is 240 × 320 except for special declaration.

For each person, from every view angle, we select the 39 frames from sequence normal-01 as the training data (gallery) and 13 frames (except for special declaration) from sequence normal-02 as the test data (probe).

For every view angle, each time we leave one training image sequence out and use the remainder as the training set. In the scenario of identification we calculate the distance between the probe corresponding to the leave out training image sequence and the 124 classes (including the leave out image sequence). In the scenario of verification, we calculate the similarity between the probe corresponding to the leave out training image sequence and the 124 classes (including the leave out image sequence).

### 4.2. The Reconstruction of Subimage

**Figure 3:**
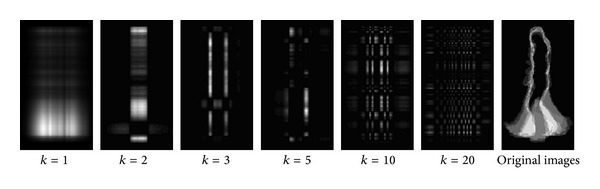
The reconstructed subimages (*k* = 1,2, 3,5, 10,20) and the original images.

Formula (17) indicates that we can reconstruct the subimage from the *W*_*k*_ and *Y*_*k*_.  Figure 3 shows the result of the reconstruction. For the consideration of illustration we normalize the brightness of every image into the range of 0–255.

As showed in Figure 3, the first and the second (*k* = 1, *k* = 2 in formula (17)) subimages corresponding to large eigenvectors of *C*_*t*_ contain the most energy of the original images. With the increase of *k*, the subimage contains more detailed information.

**Figure 4:**
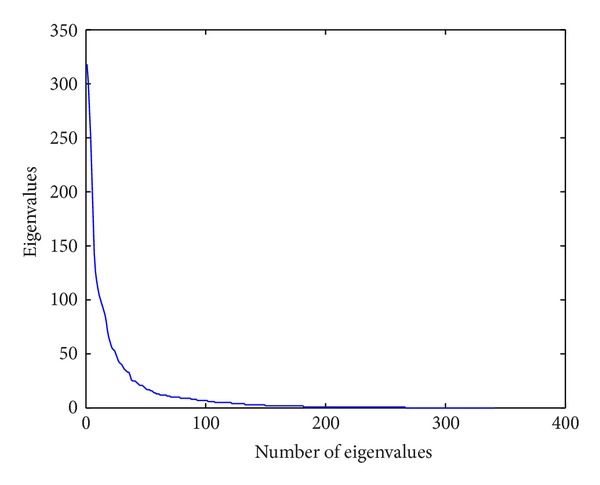
The magnitude of eigenvalue.

We also demonstrate the eigenvalue calculated by 2DPCA.  Figure 4 shows the magnitude of the eigenvalues that quickly converges to zero.

### 4.3. Performance Evaluation

#### 4.3.1. Comparison of AGDI and GEI

We compare the performance of our AGDI base method with that of gait energy image based method (In this paper, we use real template for GEI method [25].).  Table 1 shows the rank 1 and rank 5 identification rates comparing with GEI.

**Figure 5:**
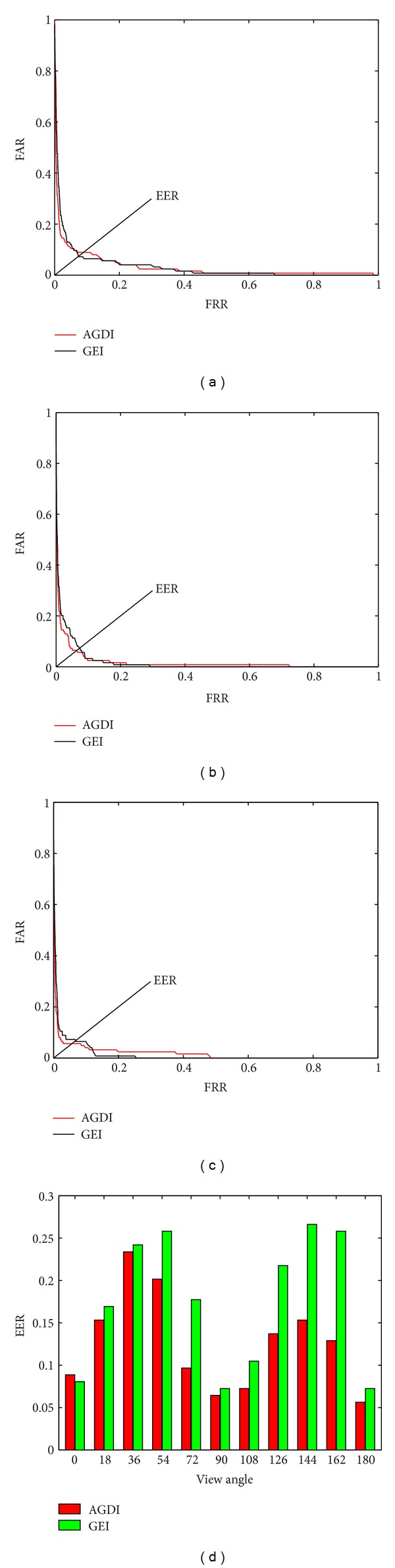
(a)–(c) The comparison of ROC curves of AGDI and GEI with view angles 0°, 90°, and 180°, respectively. (d) The comparison of EERs of AGDI and GEI with view angles 0°–180°.

To compare the performance of verification, we also evaluate the FAR (false acceptance rate) and FRR (false rejection rate) for AGDI and GEI. The ROC (receiver operating characteristic) curves under view angles 0°, 90°, and 180° are shown in Figures 5(a)–5(c). The comparison of EERs (equal error rate) is shown in Figure 5(d).

From Table 1 and Figure 5, we can see that almost under every view angle AGDI has better performance comparing to GEI (except that it is comparable under 0 view angel).

As also can be seen in Table 2, the best performance was obtained from the walking sequence taken from 0°, 90°, and 180°, while the worst was obtained from the walking sequence taken from 36° and 54°. This is probably due to the least visual deformation in the former degrees but more in the latter ones.

#### 4.3.2. The Effect of Images Amount

From the definition of AGDI (formula (3)) we can see that the AGDI image is the average value of differential images. It should be expected that the use of more images as sample would contribute to a more precise result. To demonstrate this effect, a test was conducted by selectively choosing 13, 26, and 39 (approximately corresponding to 1, 2, and 3 gait cycles) images from 90 degree in sequences normal 0l-02 as test dataset probe. Figure 5 shows the experimental result.

Indeed in Figure 5 the performance of 26 and 39 images is much better than that of 13.

#### 4.3.3. Comparison of 2DPCA and PCA

We also design an experiment to compare the performance of 2DPCA and PCA, which were applied in the step of feature extraction, respectively. The data set view angel is 90° and every frame is resized to 120 × 160.

As illustrated in Figure 7, the performance of 2DPCA, achieving the maximum at about 25 dimensions, is much better than PCA.

The key step for both PCA and 2DPCA is to get the eigenvalue and eigenvector from the covariance matrix Ct. For PCA method, every line of the covariance matrix corresponds to an image, as does the whole covariance matrix for the 2DPCA. That is, if the image size is *m* × *n*, for PCA, the covariance matrix will be an (*m* × *n*) × (*m* × *n*) matrix, while for 2DPCA it is just an *m* × *m* matrix. We resize the silhouette to different sizes and compare the CPU time of PCA and 2DPCA for the step of feature extraction.

From Table 2 we can see that 2DPCA is more efficient than PCA, especially when the image is large.

## 5. Conclusions

**Figure 6:**
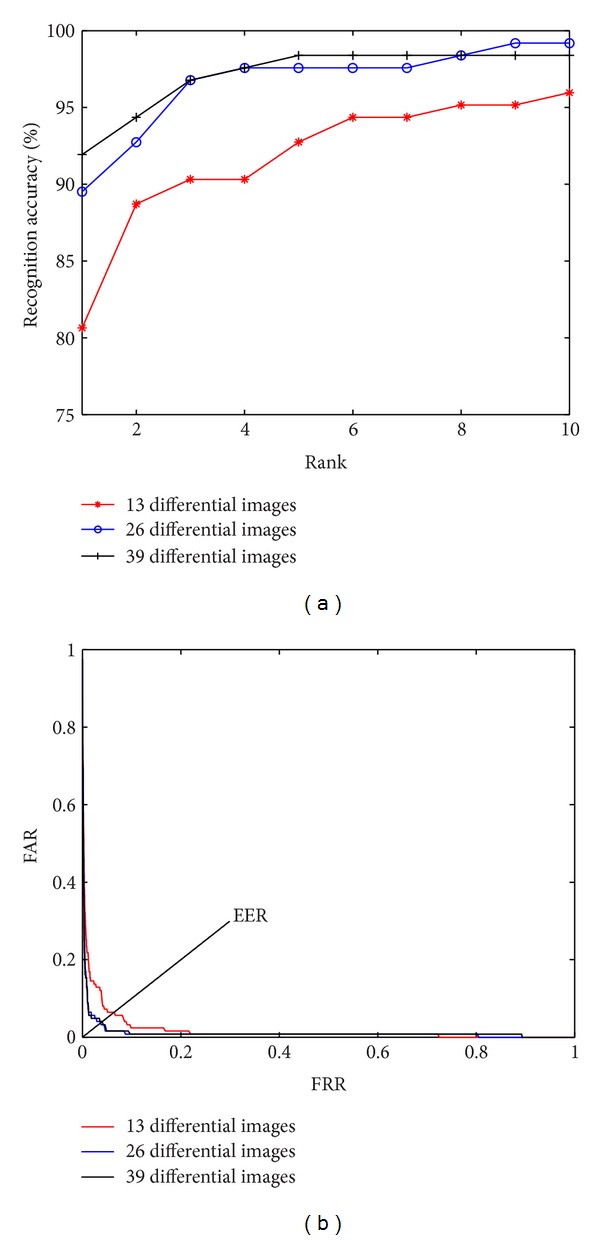
(a) Recognition accuracy of different probe sizes. (b) The comparison of ROC curves of different probe sizes.

**Figure 7:**
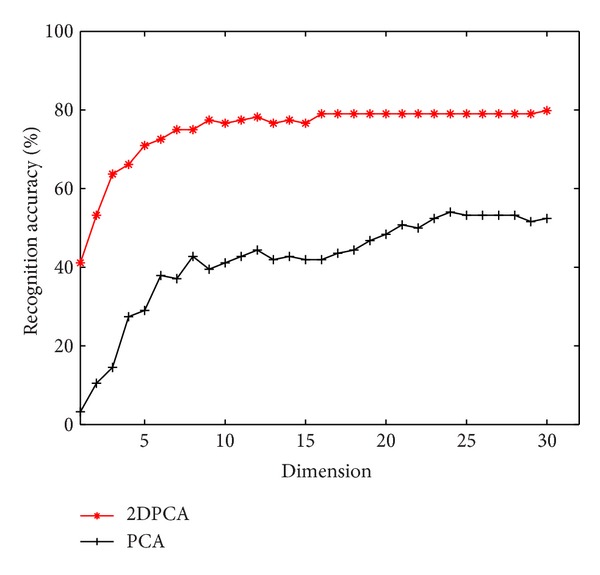
The rank 1 recognition accuracy comparison of 2DPCA and PCA.

An average gait differential image based human recognition method is proposed in this paper (Figure 6). The Kernel idea of AGDI is to apply the average of differential image as the feature image and use the two-dimensional principal component analysis to extract features. Experiments on CASIA dataset show the following. (1) Comparing to GEI, AGDI method achieves better identification and verification performance. (2) Comparing to PCA, 2DPCA is more efficient and needs lower memory storage. (3) The 0, 90, and 180 degrees silhouettes are more fit to AGDI base recognition.

## Figures and Tables

**Table 1:** Comparison of identification performance of AGDI and GEI.

View angel	Rank 1 performance		Rank 5 performance	
	AGDI	GEI	AGDI	GEI
0°	72%	68%	88%	89%
18°	54%	37%	73%	60%
36°	35%	22%	51%	44%
54°	44%	26%	55%	45%
72°	66%	44%	86%	66%
90°	81%	77%	93%	90%
108°	78%	62%	92%	85%
126°	46%	36%	73%	53%
144°	49%	34%	72%	52%
162°	59%	35%	75%	48%
180°	88%	84%	94%	93%

**Table 2:** Comparison of CPU time (ms) for PCA and 2DPCA feature extraction (CPU: Intel Core i3 2.30 GHz; RAM: 4 GB).

Feature extraction method	Image size					
	32 × 24	64 × 48	96 × 72	128 × 96	160 × 120	192 × 144
2DPCA	17 ms	47 ms	105 ms	167 ms	257 ms	431 ms
PCA	117 ms	318 ms	1273 ms	4288 ms	8896 ms	17876 ms
